# A New Perspective on Human Rights in the Use of Physical Restraint on Psychiatric Patients-Based on Merleau-Ponty’s Phenomenology of the Body

**DOI:** 10.3390/ijerph181910078

**Published:** 2021-09-25

**Authors:** Younjae Oh

**Affiliations:** College of Nursing, Research Institute of Nursing Science, Hallym University, Hallymdaehakgil 1, Chuncheon 24252, Gangwon-do, Korea; okim1108@hallym.ac.kr; Tel.: +82-33-248-2726

**Keywords:** human rights, physical restraints, mental health, Merleau-Ponty, phenomenology of the body

## Abstract

(1) Background: Physical restraint in psychiatric settings must be determined by health care professionals for ensuring their patients’ safety. However, when a patient cannot participate in the process of deciding what occurs in their own body, can they even be considered as a personal self who lives in and experiences the lifeworld? The purpose of this study is to review the existential capability of the body from Merleau-Ponty’s phenomenology to explore ways of promoting human rights in physical restraint. (2) Methods: A philosophical reflection was contemplated regarding notions of the body’s phenomenology. (3) Results: Merleau-Ponty’s body phenomenology can explain bodily phenomena as a source of the personal subject, who perceives and acts in the world, and not as a body alienated from the subject in health and illness. Patients, when they are physically restrained, cannot be the self as a subject because their body loses its subjecthood. They are entirely objectified, becoming objects of diagnosis, protection, and control, according to the treatment principles of health care professionals. (4) Conclusions: The foundation of human rights, human being’s dignity lies in the health professionals’ genuine understanding and response to the existential crisis of the patient’s body in relation to its surrounding environment.

## 1. Introduction

*“Look here, look at this bruise. Maybe it is because I was wringing my hands hard trying to get out…it hurts. I get angry. This is unfair (looking at the bruise)…every time I see this bruise, I remember the shocking moment. What could I do?”*

*“It feels like I was about to be crucified. They tied my hands and feet apart to the corners, and I could not budge.”*

The interviews above were excerpted from a phenomenological study on psychiatric patients’ experience of physical restraint, which explored what they remembered and how they felt when they had both their arms and legs tied down in a “four-point restraint” position [1]. These are stories about the experience of psychiatric patients, who are also our contemporaries and members of our community. They, who are also our neighbours, suffered from a wide range of painful conditions. Physically, these conditions included bruises, oedema, abrasions, redness, pressure sores, muscle weakness, thrombosis, and dyspnoea from not being able to move for a long time. Thrombosis or dyspnoea may even lead to death [2]. Psychologically, these patients experienced delusions or hallucinations and also emotional responses such as helplessness, frustration, anxiety, and anger [3]. Post-materialistic and communitarian values in modern society emphasise human dignity and basic human rights, patients’ self-determination and right to know, and solidarity.

Physical restraint in the mental health care setting refers to the practice of restricting the body movement of patients to prevent them from causing harm to themselves or to others. Therefore, physical restraint is considered inevitable if it is used as a last resort to protect the safety of patients and others in mental health care [4]. In particular, physical restraint used in psychiatric patients involves tying the wrists and ankles of a patient to their bed with straps or leather restraints [5]. The purpose of physical restraint is to “protect” and “control”. As mentioned above, physical restraint is used to protect the patients themselves as well as others, but inevitably becomes a tool restricting the free will of psychiatric patients––thereby compromising their basic human rights. In other words, the application of physical restraint for safety, not only generates physical and psychological problems in patients but also poses an important question about the value-judgement regarding the dignity and autonomy of human beings with distinctive and unique personalities. Since the “health and safety” of the patients and their “autonomy and dignity” are important parameters of their basic human rights—which must be ethically protected in the sense of oughtness, it is necessary to strike a balance between them, instead of choosing one over the other. Although currently, the most frequently used approach when an ethical problem occurs in health care settings is Beauchamp and Childress’ principlism [6], the limitations of its application have been continuously debated. For instance, even if the use of physical restraints is justified by Beauchamp and Childress’ principlism, physical restraints should be applied carefully. Because this principlism focuses on decision-making about life and death considering the clinical context, the patients’ social context (e.g., individual preferences, personal relationships, or economic conditions) is hardly taken into account in the decision [1,7]. 

As many countries are likely to violate human rights, they specifically present laws, regulations, and guidelines related to physical restraint as minimum protective measures to protect fundamental rights. The role of mental health professionals is not just to strictly implement physical restraint to meet legal standards. Mental health professionals should care for patients as holistic human beings and promote their physical and psychological well-being before and after the restraint decision in the entire process of physical restraint, and consider ethical issues related to physical restraint as advocates and moral agents. In particular, in order to protect and promote the human rights of patients as much as possible, it is necessary to protect the personhood of patients with ambiguous boundaries due to mental illness. Phenomenological studies on people with mental illness who have experienced physical restraint have urged nurses and physicians to understand the loss of the subject and their devastating suffering from the loss of personhood [3,8]. These patients’ demands (or voice) are consistent with person-centredness in the mental health paradigm for long decades—to see the person’s world through their eyes and listen to their story. In fact, the person-centredness of psychiatry is deeply related to Carl Rogers’ “person-centred” therapy, which originated in the phenomenology of the body [9]. Therefore, mental health professionals have a duty to protect and promote human rights by recognising the dignity of vulnerable people in distress while being physically restrained.

In his phenomenology of the body, Merleau-Ponty emphasises the concept of the embodied self as the foundation of the personal self, who lives in the lifeworld and experiences it. He describes the embodied self as a willing subject who perceives the world and rises towards it in terms of body-intentionality [10]. His phenomenology does not describe the body, served as an object of medical diagnosis and treatment, and alienated from the personal subject; but instead records the phenomenon of the body as a source of the personal subject that perceives, desires, and acts in this world. Therefore, Merleau-Ponty’s phenomenological perspective on the body can facilitate a better understanding of the physical phenomena of an individual damaged from illness and the diverse approaches of care that can help the patient’s body reside in the lifeworld. 

Therefore, the purpose of this study is to critically consider the limitations of principlism to justify the use of physical restraint and to review the existential capability of the body from Merleau-Ponty’s phenomenological perspective, reflecting critically psychiatric patients’ experience of physical restraint in the process. Ultimately, this study could present a new perspective on human rights to health care professionals caring for physically restrained patients in psychiatric settings.

### Human Rights Regarding the Use of Physical Restraint in South Korea

Since the Mental Health Act was enacted in December 1995, South Korea has begun to take an aggressive interest in mental health-related human rights, somewhat later than other countries [4]. The Mental Health Act has been revised several times, and the government and society have continued to show interest in the rights of people with mental illness, nonetheless, recently, articles on human rights violations related to physical restraint of psychiatric patients are also being constantly reported [11,12].

According to a nationwide survey conducted by the National Human Rights Commission of Korea, in 2015, isolation and constraint measures were used in 56.3% of the 500 patients admitted in national, public, and private mental health institutions across the nation. They were physically restrained three times on average, and 11% were restrained more than 10 times. Most patients reported being restrained for 3 h or longer (71.9%), and 10.9% of them were even restrained for 24 h or longer. A total of 24.9% of the seclusion and restraint cases were the result of violations of ward regulations. The use of physical restraint varies among facilities depending on the physician or the psychiatric setting, although the reason might be the same, which is the possibility of the patients inflicting harm on themselves or others [5]. That is, physical restraint could be determined differently according to who the physicians are or where the psychiatric settings are. In response, the Korean government wholly amended the Mental Health Act in May 2016 to revise the provisions on seclusion and restraint and provide specific guidelines, consisting of the definition of seclusion and restraint, application criteria, principles of application, and their implementation log forms. A year later, in 2017, the Mental Health Act was amended again. Article 75 (Prohibition of Isolation or other Restrictions) made the requirements for the use of physical restraint more demanding by mandating the use of seclusion and physical restraint for medical purposes only by psychiatrists [13]. To promote the human rights of psychiatric patients, the head and workers of each mental health facility have been mandated to study human rights for at least 4 h every year since 2009 (Article 70: Human Rights Education) [13].

However, a study of mental health workers in Korean psychiatric hospitals showed that mental health workers have a low level of human rights sensitivity in the use of physical restraint [14]. Some point out that the government’s human rights education system should comprise an in-depth focus on real human rights than on punishment and regulation [15]. Additionally, in terms of interpretation and application, active consideration is needed towards rights and legal aspects. The specific criteria are unclear, and there is no delegation requirement to subordinate statutes to establish specific criteria. Several scholars argue for the need to actively promote human rights in the area of physical restraint [4,16].

## 2. Limitations of Principlism to Justify the Use of Physical Restraint

Principlism designates an approach to biomedical ethics that uses a framework of four universal and fundamental ethical principles: respect for autonomy, nonmaleficence, beneficence, and justice. Principlism, combined with the Hippocratic Oath or Christian ethics—which are based on natural law and the deontological and utilitarian norms of ethics—constitute medicine’s ethical principles [17]. Based on principlism, nurses or physicians regard ethical issues—such as the patient’s autonomy, informed consent, life support, patient confidentiality, termination of meaningless life-sustaining treatment—as a problem situation that should be addressed according to ethical principles. This problem-solving requires a process of ethical reasoning through prioritising and striking a balance between principles. Health care professionals observe an impersonal, neutral, and detached attitude in accordance with these principles. Thus, healthcare professionals are drawing upon principles that are limited, although this does not rule out that some or many of them also take these issues into account. The principles may of course discourage the consideration of individual differences in patients and families and the unique experience of illness of each patient [18,19].

Whether the patient can threaten the safety of others and themselves should be decided based on the consideration of all four foundational principles of principlism. Nonmaleficence would entail not inflicting harm by using restraints, and beneficence would mandate making the best choice that would contribute to the welfare of both the patients and others. Justice would require the safety of the patients and others to be valued equally, and respect for autonomy would demand that patients be provided with the autonomy to make choices pertaining to their own bodies. The principles of nonmaleficence, beneficence, and justice imply ethical oughtness that protecting lives and safety must come before any other consideration in making ethical decisions, even though respect for autonomy should be considered, thereby ensuring the ethical validity of using physical restraint [1].

The limitation of principlism is that it does not take into account the patient’s personal experience on physical restraint, even though it violates human rights. The overuse of physical restraint due to paternalism can be the fallacy of a slippery slope, which generates serious violations of human rights [3,20]. Of course, although it is legally agreed upon by the patient before physical restraint is applied, principlism has little perspective on truly comforting and understanding the patient’s dignity and human rights in the process of determining and caring for patients. Patients who are physically restrained are human beings with their own unique personalities. Respect for persons is a core value of human rights; personality is accorded the highest ethical status, and a decision cannot be considered ethical if it invalidates a person’s free will [17]. From the patients’ point of view, physical restraint is an experience of being deprived of the right to make autonomous decisions regarding their own body—thereby subjecting them to decisions based on the judgement of others and rendering them isolated from the outside world and painfully vulnerable. Thus, when a patient cannot participate in the process of deciding what happens to their own body, can they even be considered as a personal self who lives in and experiences the lifeworld where they dwell? As Heidegger said, human beings’ dwelling means that they take care not only of their own life but that of other beings. In other words, principlism cannot be free from the criticism that unconditional application of ethical principles does not consider the unique positions and interests of individuals in the concrete context of life.

Moreover, concerning justice, it can cause inequity in the real world because the ethical concepts that constitute principlism presuppose values based on different ethical beliefs. The conflict between principles results from the fact that these ethical concepts—respect for autonomy, nonmaleficence, justice, and beneficence—are grounded in different ethical worldviews. Thus, nurses or physicians inevitably make different ethical decisions based on their preferred ethical views and principles [21,22]. The existential situation and the difference in cases are analysed mainly by health care professionals. In the real-world, physical restraint has been found to be applied disproportionately to patients with mental illness. Up to 90% of those restrained in the emergency room were psychiatric patients [23,24], while 33.7% of the analysed restraint cases involved substance abuse [23,25]. Hall and Smithard [7] pointed out that more frequent application of physical restraints to a particular group based on physician’s prejudice contradicts the principle of justice because it is an inequitable treatment. 

Under these circumstances, it must be considered whether health care professionals see the body of a physically restrained patient as an object or a subject. They should consider their patients as “unique autonomous persons” and thereby develop a new perspective on human rights regarding the use of physical restraint. I have explored this question based on Merleau-Ponty’s body phenomenological perspective.

## 3. Merleau-Ponty’s Body Phenomenological Perspective

### 3.1. Body as the Subject of the Illness Experience

In his Phenomenology of Perception, Merleau-Ponty sees the body as an embodied subject having intentionality, which is directed towards the world. Merleau-Ponty explains the habitual body as being of general and pre-reflexive existence, distinguishing it from the actual body as being of personal and reflexive existence. Co-penetrations between them always occur [10]. According to Merleau-Ponty, our body takes a structured form in a specific way when communing with, perceiving, and acting towards the world, and this is called the body schema. This body schema is fixed in the body through repetitive actions, which constitute the habit-body [26]. Therefore, having bodily habits signifies that we live, engage in, and experience the world in a particular way through the body. For example, we are able to live our daily lives and perform actions according to our situation through motor habits formed in the body. The habit presumes a form of “understanding” that the body has of the world where it carries out its operations. As Merleau-Ponty explains, habit bears a direct relation to this form of a dialogue between environment and subject. Its role is to establish in time those behaviours or forms of conduct that are appropriate for responding to the invitations of the environment [27]. There is no form between the movement of the body and the world, but rather the body “adapts” to the invitation of the world [10]. Thus, we culturally acquire the habit of using new tools to complete a task, either using their own natural body or through general tools, like a cane or glasses [28]. Such habits form implicit knowledge that already exists in our body—embedded in our hands, eyes, ears, arms, and legs [17].

Merleau-Ponty talks about the bodily existential capacities to engage in concrete situations based on the habit-body. He refers to the “I can” capacity of integrating bodily perception, movement, and intentions as the “intentional arc”, which is “certain concrete freedom, the general power of placing oneself in a situation when tied to actuality” [29]. The “intentional arc” capacity is the bodily existential capacity to engage in actual situations, uniting all the senses, ideas, and bodily movements [17]. Thus, it emerges from the body’s freedom. When we are ill and have severely inhibited bodily capacities, we are placed in an unfamiliar world, experiencing pain as our body fails to meet our feelings and desires. This is because the bodily capacity to engage in and communicate with the surrounding world has been disabled [30].

As discussed above, Merleau-Ponty’s bodily experience—of engaging in the world following the body schema unconsciously—reflects the old habits, intentions, and attitudes formed in our daily lives; that is, our capacity to participate in situations. For instance, the bodily experience of the psychiatric patients who are physically restrained, with all four of their limbs tied down, represents their frustration with a life where they cannot engage in the world as a subject of desire. Therefore, the process of the recovery of bodily habits (bodily situations)—in which the body can engage in situations with familiarity—is associated with the process of the existential engagement of the body, during which the perception, intentions, and desires of an individual are projected onto their situations [31]. For example, when intensive care unit patients are discharged after a long hospitalisation, they experience bodily responses in the process of recovering their bodily capacities through physical contact with an environment full of familiar and habitual bodily knowledge. This demonstrates that bodily existential capacities can recover in a familiar environment full of one’s habitual knowledge and incarnated meanings. When we are healthy, we do not pay attention to our bodily phenomena, but when we suffer from illnesses or injuries, we attend to our body and acquire phenomenological skills. When we feel physical pain, shortness of breath, or impaired mobility, we experience bodily difficulties as existential crises.

In this respect, the phenomenological description of psychiatric patients’ bodily experience of physical restraints could provide a deeper understanding regarding how bodily capacities required to live in the lifeworld can be disabled and how this poses an existential threat to the individuals.

### 3.2. Psychiatric Patients’ Bodily Experience of Physical Restraint

According to one study on illuminating the lively experiences of physical restraint among psychiatric patients, they depicted their body encounters and struggles as being horribly disconnected from the world. One of them said, “I was afraid I would not be able to do anything if something happens while I was restrained. The tranquillisers they gave me while I was restrained made me feel hazy and drowsy, and I sang a song and screamed in order to stay awake, but the practitioners gave me more medicine and kept me restrained for longer, saying I was aggressive and uncooperative and the only thing I could think of was that I was tied up. I felt like I was dying” [32].

Patients realised that they were trapped in their bodies without being able to physically respond to what was happening around them even if they could still see, hear, and interpret the world. Patients were most perturbed by the notion that they could not escape from the situation. They were entirely objectified, becoming objects of diagnosis, protection, and control, according to the treatment principles of health care professionals [33]. Their body, as the subject of experiences—seeing, hearing, feeling, and interpreting—was excluded.

The body of a patient perceives this lifeworld as an existential subject that communicates with and resides in this lifeworld with familiarity, and a desiring subject that perceives and acts towards this lifeworld, forming the horizon in their lives [31]. A patient’s body consists of not only physical elements such as bones, flesh, and blood but also their entire experiences and perspectives directed towards the world and the foundation of the self––based on which their intentions are implemented [34]. A patient, when physically restrained, cannot treat the self as a subject anymore because his or her body loses its embodied subjectivity by the actions of others [35]. Merleau-Ponty’s concept of the “body-subject” implies that a human person is an essentially embodied being, who can interact with and find significance in his or her world only because acts of perception arise from the “body-subject” through the structures of the human body [36]. The body of a patient, even when physically restrained, is the subject of experience, which can perceive, desire, and act in this world. They, with all four of their limbs tied down, express their frustration with a life where they cannot engage in the world as a subject of desire. They experience an existential crisis where they can no longer participate in or live in the familiar lifeworld, posing a threat to the continuation of life as they have lived. In short, a patient undergoing such objectification through isolation and physical restraint is consequently experiencing entrapment in a useless body, which sees, hears, and feels, but cannot communicate with the world. Health care professionals should understand the lived body as a source of personality that experiences the lifeworld—which has often been overlooked—before physically restraining patients.

According to the phenomenological study on psychiatric patients’ experiences of physical restraint, patients reported the following about their experience: “No matter how hard I cry, no one comes to help. The more I cry, the crazier I must look”; and “I thought I would be released sooner if I stayed quiet”. At the same time, however, patients keep struggling to survive, mustering the courage to address the chaos and difficulties they encounter, and gathering the strength to keep living. To combat the uncertainties of life and their anxiety regarding the unknown, they muster all the physical and mental strength they can access. The following example clearly illustrates this process: “I tried to calm down although I was tied down. The harder I tried to untie it, the tighter the strap felt, and it badly hurt my wrists. I thought about why they tied me down. They probably did it for me. So I thought, ‘could it be part of the treatment?’” [1].

As shown in the responses above, patients continue to try communicating with the world, feeling and thinking through the body even when the body is objectified by the health care professionals through physical restraints. They view it as a bodily experience, inseparable from their perception, influence, power, and intentions, but the world becomes unfamiliar, and they feel pain over the loss of their bodily autonomy. This is a significant threat to the embodied subject, which has intentionality directed to the world. They are in the process of losing themselves as human beings. Thus, as advocates and moral agents, health care professionals must take into consideration the phenomenology of the body to care for patients in the existential crisis while maintaining and promoting their dignity, which is the central value of their human rights.

## 4. Discussion

This paper has reviewed the existential capability of the body from Merleau-Ponty’s phenomenological perspective, reflecting critically psychiatric patients’ experience of physical restraint, to present a new perspective on the human rights of psychiatric patients who are physically restrained. Notably, the examples mentioned above remarked how health care professionals should pay attention and respond to the patients’ bodily experiences, as an existential crisis, using a first-person perspective, thereby helping the patient to contact bodily familiar environment [22,31]. The health care professionals’ attitude can reveal the patients’ lifeworld and the meaning of the entire clinical situation [37]. Also, health care professionals can understand the essence of human rights while interacting with and listening to the patients carefully, focusing on a lived body [17].

This finding is supported by empirical research; the following is an excerpt from an interview with a psychiatrist at a university hospital on the use of physical restraint: “Many residents work really hard. They talk to the patients face-to-face all night, trying as hard as possible to calm them and not apply physical restraint. Their effort decreases the number of prescriptions for the use of physical restraint” [1]. Their attitude could be in accordance with the notions of a phenomenological approach in that they practise the ethics of response by listening to patients, actively showing interest in their demands, and trying to understand them [17]. The correlation between the attitude of the physicians and nurses and the use of physical restraint is evident in patients’ remarks such as, “If the nurse or the doctor had talked to me one more time, I would not have been tied down”; and “I just wanted to talk about why I was so angry and wanted them to listen to me” [32]. In Wilson et al.’s recent qualitative study [8] comprising interviews of 13 mental health inpatients and 22 staff members, both the parties commented that restraint was not used as a last resort, as more could have been communicated in order to prevent restraint. Not only healthcare professionals but also other staff, such as security guards or nurse aides, should be trained in specialised programs on communication with psychiatric patients and empathetic understanding of physically restrained patients. 

Nevertheless, some staff viewed the use of physical restraint as punishment for patients’ aggressive behaviours instead of listening to their inner voices in dealing with psychiatric patients with aggressive behaviours when they want to ask for help or interact with staff more [38]. Using physical restraint as punishment no longer takes into account everyone’s safety but is rather a violation of the patient’s human rights. Therefore, human rights education for all staff in psychiatric environments should include why, what, and how to interact and communicate with their patients with phenomenological attitudes. For instance, essays by anonymous patients on physical restraint could be used as study materials. Future research is required to explore lived experience among physically restrained psychiatric patients using phenomenological study. This finding could suggest more suitable ways to improve human rights in the use of physical restraint in psychiatric care settings. In psychiatric settings, some level of ethical competence is required for health care professionals. For instance, the phenomenological understanding of the patients’ situation and improving their moral sensitivity regarding physically restrained patients’ sufferings and pain should be more emphasised. 

Meanwhile, an approach based on the phenomenology of the body can accomplish equity among patients since every health care professional can provide consistent care according to the patients’ individual experience, rather than by experts’ beliefs or values. Besides, this new perspective can enable health care professionals to consider the issue of inequity among patients depending on their situation regarding the treatment of physical restraint. Previous findings support this. One of the common reasons for the use of physical restraint is staffing shortages in psychiatric care settings [38,39]. For instance, when several patients are at risk of harming themselves or others in a closed psychiatric ward, but the number of staff is not enough, physical restraint has been reported to occur more frequently and for a longer duration [38]. That is, the use of physical restraint depends on situations, not patients. The issue of inequity can be reflected in another context as well.

Hypothetically, there are two patients, A and B, who have been diagnosed with the same mental disease and display a similar risk of harming themselves but are under treatment in two different facilities. Patient A harms himself and experiences physical restraint frequently because he has no access to proper treatment or care in a relatively physically and psychologically flawed facility. In contrast, Patient B receives proper treatment and care in a good facility (e.g., nurses provide proper care after talking to the patient face-to-face) and therefore experiences a minimum level of physical restraint. We thus need to consider why Patient A is subjected to more physical restraint.

In most countries, the use of physical restraint is permitted by the law in situations where the patient’s behaviour poses an imminent danger of physical harm to them or others [3]. Physical restraint is therefore applied to Patient A in pursuance of the law to ensure everyone’s safety. At the same time, considering Patient A is entitled to the same medical benefits as Patient B, the use of physical restraint should be minimised. In other words, we should focus on the quality of health care in applying physical restraint on psychiatric patients. Comparing the rate of physical restraint use among psychiatric patients shows that physical restraint is used far more frequently in developing countries than in developed countries [40,41,42]. Thus, this issue of health care inequity and the infringement of individuals’ freedom should be carefully considered in the use of physical restraint.

In order to mitigate inequity caused by external conditions, a continuous education program on physical restraint for staff could be proposed as an alternative. Cases have been reported where the application of the physical restraint has resulted from problems on the staff’s end, such as their lack of knowledge [43,44]. However, the use of physical restraint due to a lack of knowledge can be preventable. Psychiatric patients consistently demonstrate prodromal symptoms, which is unusual behaviour such as irritability, anger expressions, and verbal aggressiveness, for a while before the outbreak of violence. With explicit knowledge about prodromal symptoms, staff can prevent the patients’ violent behaviour by identifying the early signs of the prodrome and listening to what the patients have to say [43,44].

Although this present study focused on the human rights of psychiatric patients, it should not be overlooked that patients’ unexpected violent behaviour can pose a severe threat to the safety of others in clinical settings, including other patients, and health care professionals [45,46]. Thus, it is necessary to provide support services for health care professionals’ life and safety as well.

## 5. Conclusions

This paper contemplates the use of physical restraint on psychiatric patients from the perspective of Merleau-Ponty’s phenomenology of the body. Merleau-Ponty’s phenomenology of the body reminds us of a human being’s dignity by focusing on the orientation of the body of the physically restrained patient, revealing the body as the subject of the self, a unique individual. To protect the fundamental human right, the right to dignity, health care professionals should notice and adequately respond to the collapse of the existential life of their patients, with an understanding about those who suffer from physical difficulties and misery, before tying them down in a secluded room. For facilities and hospitals, it is required to establish a care and support system that provides safety and comfort to patients, enabling them to realise their physical capacities and power in their situations and trust their own bodies. Lastly, institutions should continue to educate people regarding human rights based on the body’s phenomenology during the use of physical restraint in psychiatric care settings.
